# Relationship between Antioxidant and Anticancer Activity of Trihydroxyflavones

**DOI:** 10.3390/molecules22122169

**Published:** 2017-12-07

**Authors:** Ignas Grigalius, Vilma Petrikaite

**Affiliations:** Department of Drug Chemistry, Faculty of Pharmacy, Lithuanian University of Health Sciences, LT-44307 Kaunas, Lithuania; ignasgrigalius@gmail.com

**Keywords:** trihydroxyflavone, flavonoid, antioxidant, anticancer, structure-activity relationship

## Abstract

Plant polyphenols have been highlighted not only as chemopreventive, but also as potential anticancer substances. Flavones are a subclass of natural flavonoids reported to have an antioxidant and anticancer activity. The aim of our study was to evaluate antioxidant and anticancer activity of seventeen trihydroxyflavone derivatives, including apigenin (API) and baicalein (BCL). Also, we wanted to find out if there is a correlation between those two effects. Cell growth inhibition testing was carried out using MTT assay in three different human cancer cell lines: lung (A549), breast (MCF-7) and brain epithelial (U87). Antioxidant activity was determined by the DPPH radical scavenging method. Thirteen trihydroxyflavones possessed anticancer activity against at least one tested cancer cell line. They were more active against the MCF-7 cell line, and the lowest activity was determined against the U87 cell line. The majority of compounds inhibited cancer cell growth at EC_50_ values between 10–50 µM. The most active compound was 3’,4’,5-trihydroxyflavone **7**, especially against A549 and MCF-7 cell lines. The correlation between anti-proliferative and antioxidant activity was only moderate, and it was determined for A549 and U87 cancer cell lines. The most important fragment for those two effects is the *ortho*-dihydroxy group in ring B. Conclusions. Trihydroxyflavones demonstrated anticancer activity. Further and more detailed studies should to be carried out to estimate the structure–activity relationship of these compounds.

## 1. Introduction

Cancer is one of the major causes of mortality worldwide. Despite tremendous efforts to create effective chemotherapy drugs, there is still a huge toxicity and selectivity issue. The toxicity of modern chemotherapy and cancer cell resistance to anticancer agents leads us to seek new treatments and prevention methods of this insidious disease [1,2]. The importance of plant substances in medicine and pharmacy is well known from ancient times; herbal substances are often used as the basic structure in the development of new anticancer drugs [3]. In the last 20 years, more than 25% of new drug molecules were directly obtained from the plant sources, and another 25% were chemically modified herbal substances [4]. About half of drugs approved from 1994 to 2007 were of natural origin [5]. Also, herbal remedies and their derivatives strongly contribute to our understanding of the mechanisms of cancer development.

However, regardless of the intensive research on plant material, the biological activity is known for less than 15% of plants, and they remain an attractive source for scientists to find new molecules. Natural anticancer drugs have a low cost and possible several mechanisms of action [6], and they are often effective against chemotherapy resistant cancer cells. Nowak highlights the importance of chemotherapy or radiotherapy used together with polyphenol compounds [7].

**Figure 1:**
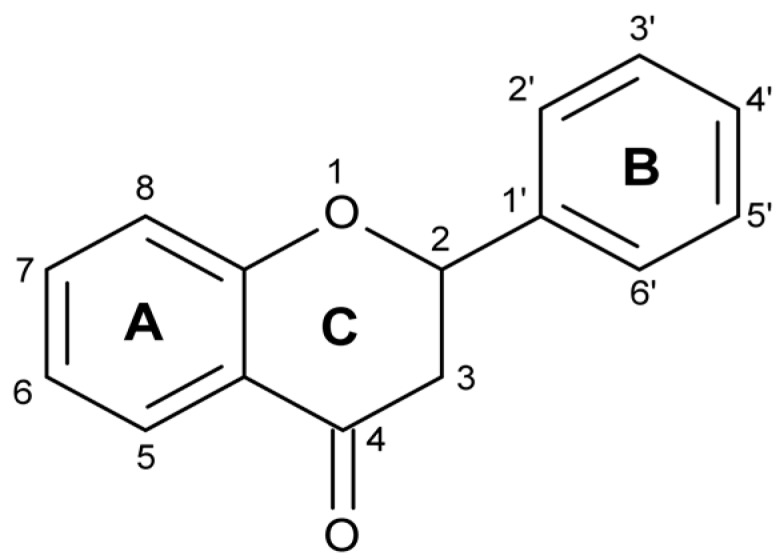
General structure of flavonoids.

Flavonoids are one of the most tested and widely distributed substances of plant origin [8,9]. They are found in fruits, vegetables, leguminous plants and even some kinds of moss [10]. The skeleton of flavonoids consists of 1-benzopyran. It is a C6-C3-C6 system (Figure 1), in which aromatic rings A and B are connected through ring C, forming a central pyran or pyron cycle [11,12,13]. Depending on the position to which ring B is connected to the chromane ring, flavonoids are classified into isoflavonoids and neoflavonoids [14,15]. Usually, flavonoids contain hydroxyl groups in positions 2, 3, 5, 7, 3’, 4’ and 5’ [16]. Ring A most often has hydroxyl groups in position 5 and 7, and ring B in position 4’ (if one hydroxyl group), or 3’ and 4’ (if two hydroxyl groups), or positions 3’, 4’, and 5’ (if three hydroxyl groups) [17].

Flavones are one of the largest flavonoid subclasses. They contain a double bond between C2 and C3 in heterocycles, as well as a C4 carbonyl group. Benzene ring B is connected to the C2 atom in central chromone cycles. Usually, flavones do not have substituents in the C3 position [10,18,19]. Most common natural flavones are luteolin and apigenin (API) [18]. A very important structural feature of flavones is the arrangement of hydroxyl groups, which determines their activity, especially anti-proliferative and kinase inhibiting effects [12].

Flavones possess a wide range of different biological activities. They act as antioxidants, inhibit cell proliferation, and have antimicrobial, estrogenic, anti-inflammatory effects [20]. Flavones participate in binding the reactive forms of oxygen or nitrogen, possess activity against human immunodeficiency virus, lower lipid levels in blood, act spasmolytically, dilate blood vessels, and inhibit thrombus formation [20,21]. Also, those compounds participate in cellular molecular mechanisms related to cancer occurrence and development; thus, they might be used not only for cancer prevention, but also as anticancer agents [12]. However, the anticancer mechanism of action of flavones is not very clear. It was established that they could inhibit cancer cell proliferation, differentiation, induce apoptosis, and interfere in angiogenesis, inflammation, and inhibit metastasis [22].

API and baicalein (BCL) are the most widely studied trihydroxyflavones. It was established that those compounds may inhibit cancer cell proliferation [1,12]. BCL affects lung and breast cancer cell proliferation and does not exert any effect on normal cells [23]. API inhibits MCF-7 cell proliferation by inducing cell apoptosis [24]. In the study conducted by Marder [25], API together with a synthetic nitro group containing flavones was one of the most active inhibitors of proliferation. API also inhibited the proliferation of cervical, lung, hepatoma, and bladder cancer cells [26,27].

It is supposed that flavone cytotoxicity is related to the hydroxyl group number and position in their structure [28]. Pouget et al. [29] studied the influence of substituents in ring A of flavanones, chalcones and flavones on the proliferation of the MCF-7 breast cancer cell line. Non-substituted flavone and monohydroxylated flavones possessed low anticancer activity, while 7,8-dihydroxyflavone was the most active of all tested compounds. Kawaii et al. [30] proved that the double bond C2–C3, *ortho*-catechol group and C3 hydroxyl group are important for anti-proliferative activity. Surprisingly, all tested flavonoids did not possess any effect on normal human cell viability.

The antioxidant activity of flavonoids is studied quite widely. They disrupt the electron transport chain due to their radical scavenging properties and also chelate metal ions [16]. The antioxidant activity of hydroxyflavones depends on the number of hydroxyl groups and their position in the molecule. Hydroxyl groups in ring B are very important for binding hydroxyl, peroxyl and peroxynitrile radicals [16]. 

Unsubstituted flavone and monohydroxylated flavones do not possess antioxidant properties [31]. However, dihydroxyflavones, especially those containing *ortho*-dihydroxy group, are strong antioxidants. Trihydroxyflavones BCL and galangine lack this structural element and have 2.5–5 times less activity. API does not possess DPPH radical-scavenging properties. 

Hyun et al. [14] found that the free radical scavenging effect increases with an increase in the number of hydroxyl groups in the flavonoid molecule. Higher antioxidant activity was observed for those compounds that contained *ortho* hydroxyl groups. Other structural elements are important as well: C2–C3 double bond and C4 keto group, hydroxyl groups in positions 3 and 5 [22,32]. Gomes [33] compared the antioxidant activity of trihydroxyflavones containing hydroxyl groups in different positions. It was established that the *ortho*-dihydroxy group is required for free radical scavenging activity.

Despite the numerous studies of separate flavonoids and plant extracts enriched by those compounds, the relationship between anticancer and antioxidant activity remains unclear and still has to be studied more thoroughly.

Hydroxyflavones are very attractive compounds for anticancer activity studies since they have low toxicity and may interact with DNA [22]. Flavopiridol is a synthetic derivative, similar to flavonoids. It is known that this compound has an effect on cyclin-dependent kinases [34]. It has been tested in clinical trials as a drug from chronic lymphocytic leukemia [21,35]. This example proves the importance of natural substances, such as flavonoids, in the development of new drugs. Better understanding of structure–activity relationship of tricycle phenolic compounds could contribute to the discovery of new generation anticancer compounds.

## 2. Results and Discussion

### 2.1. Anti-Proliferative Effect

**Figure 2:**
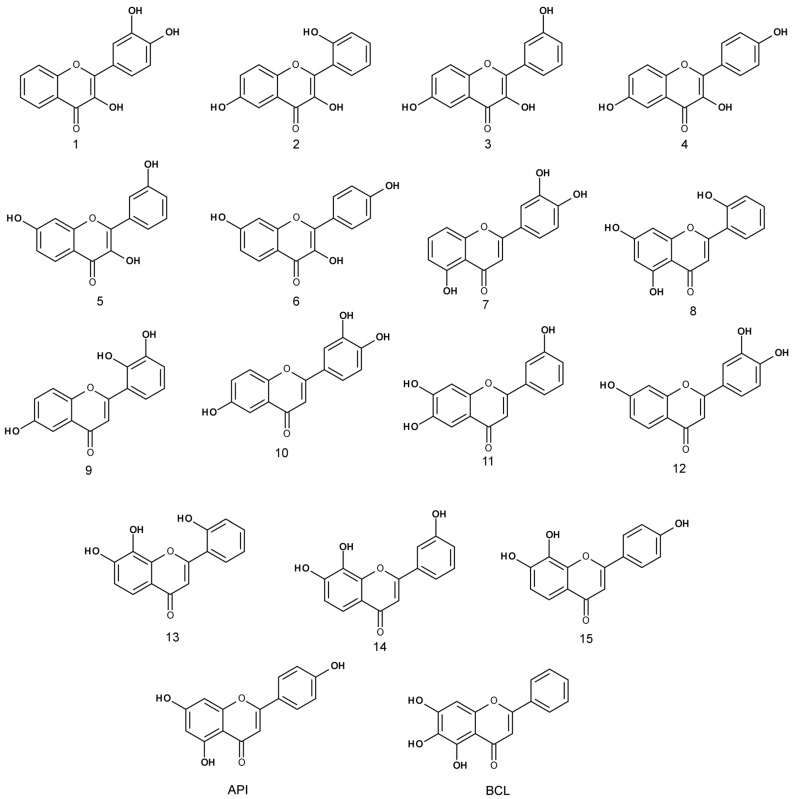
Chemical structures of tested trihydroxyflavones.

The anti-proliferative activity of trihydroxyflavones (Figure 2) possessing hydroxyl groups in different positions of 2-phenyl-1,4-benzopyrone was tested in three cancer cell lines: human non-small cell lung carcinoma (A549), human breast adenocarcinoma (MCF-7) and human glioblastoma (U87).

The trihydroxyflavone effect on cancer cell viability was variable (Table 1). Thirteen out of seventeen tested trihydroxyflavones possessed cytotoxic effect at least against one cancer cell line. Compounds were mostly active against the MCF-7 cell line, and possessed the lowest activity against the U87 cell line. Only two compounds (**1** and **3**) showed anti-proliferative effect against glioblastoma cells at lower than 25 µM concentration. Most trihydroxyflavones inhibited cancer cell proliferation at concentrations in the range from 10 to 50 µM.

API did not possess high activity against all tested cell lines, whereas BCL was moderately active against the MCF-7 breast cancer cell line. Scherbakov et al. [36] established stronger anti-proliferative effect of API on human breast cancer cells (EC_50_ = 25 µM). Shukla [37] suggests that different activity of API in cancer cell lines depends on cell prototypes, e.g., breast cancer cells with different epithelial growth factor receptors (HER2/neu) have different responses to API treatment. In our experiments we did not see API activity in the glioblastoma cell line. However, Stump et al. [38] discovered that API reduced U87 cell viability even at lower concentrations than 100 µM. The discrepancy between results in our experiments and previous findings could be due to the differences between cancer cell lines and the slightly different experimental conditions, e.g., medium pH. BCL activity was quite modest in our experiments and those data were similar to previous findings. Yan et al. [39] established the EC_50_ for BCL against the MCF-7 cell line as 66.3 ± 5.9 µM, and Lee [40] found that BCL reduced the lung cancer cell line CH27 viability at 50 µM concentration. 

**Figure 3:**
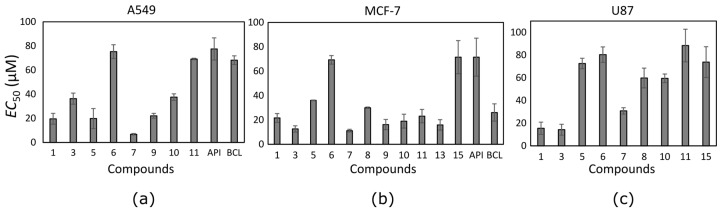
The EC_50_ values of the most active trihydroxyflavones against (**a**) A549, (**b**) MCF-7, and (**c**) U87 cancer cell lines.

Ten trihydroxyflavones inhibited proliferation of non-small cell lung cancer cells at lower than 100 µM concentrations (Figure 3a). The most active against the A549 cell line was compound **7** which contains two neighbouring hydroxyl groups in ring B. Compound **1** containing hydroxyl groups in the same positions of ring B but lacking hydroxyl groups in ring A, possessed 2.9 times lower activity. Similar activity was determined for compound **9**. Compound **10** differs from compound **9** only in the different position of one hydroxyl group in ring B, and this could determine its lower activity. Compounds **3** and **5** contain hydroxyl groups in the same positions of rings B and C; only the hydroxyl group position in ring A is different, and the EC_50_ value is twofold different (EC_50_ is 36.3 ± 4.6 and 19.8 ± 8.3 µM, respectively).

Almost all tested trihydroxyflavones (13 out of 17) inhibited the proliferation of MCF-7 cancer cells (Figure 3b). The same compounds that reduced A549 cell viability were active also against the breast cancer cell line and even showed higher activity. Compound **7** appeared to be the most active among all trihydroxyflavones. It reduced MCF-7 cell viability twofold more than that of BCL and sixfold more than that of API. The EC_50_ values of most compounds varied between 12 µM and 24 µM.

Trihydroxyflavones were not very active against glioblastoma cell proliferation. Nine out of seventeen tested compounds reduced U87 cell viability at EC_50_ values lower than 100 µM (Figure 3c). Glioblastoma cells were resistant to API and BCL. This lower activity against the glioblastoma cell line could be attributed to auxiliary transporters that are typical of brain tumour [41]. Glioblastoma is one of the most common malignant brain tumors. It is very aggressive and usually resistant to chemotherapy (e.g., temozolomide), and after diagnosis of glioblastoma, the survival time of patients is about 12–15 months [42].

**Figure 4:**
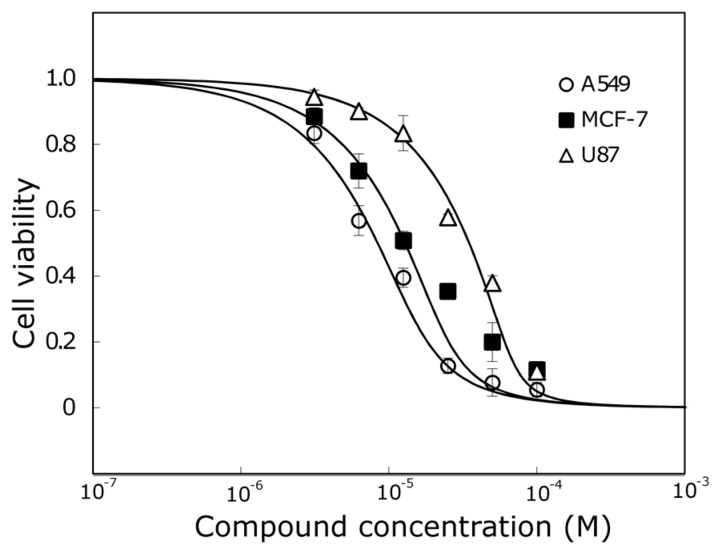
Comparison of compound **7** activity against A549, MCF-7 and U87 cell lines.

Compound **7** that was the most active against A549 and MCF-7 cell lines (Figure 4) was not the most active against the human glioblastoma cell line. Compounds **1** and **3** reduced U87 cell viability twofold more than compound **7** and were the most active compounds against glioblastoma cells. Those trihydroxyflavones possess the only hydroxyl group in the same position of ring C.

**Figure 5:**
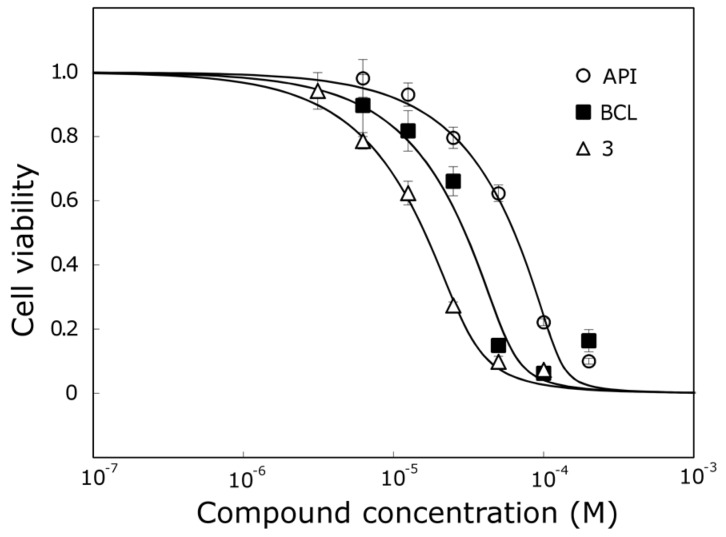
The activity of API, BCL and compound **3** against MCF-7 cell line.

Compound **3**, which was one of the most active trihydroxyflavones, was 5.7 times more active than API and 2 times more active than BCL against the MCF-7 cell line (Figure 5).

However, compound **3** does not contain the *ortho*-dihydroxy group in its structure and all hydroxyls are attached to different rings. We hypothesize that it could have a different mechanism of action than other trihydroxyflavones, and more detailed anticancer studies are needed

### 2.2. Antioxidant Activity

**Figure 6:**
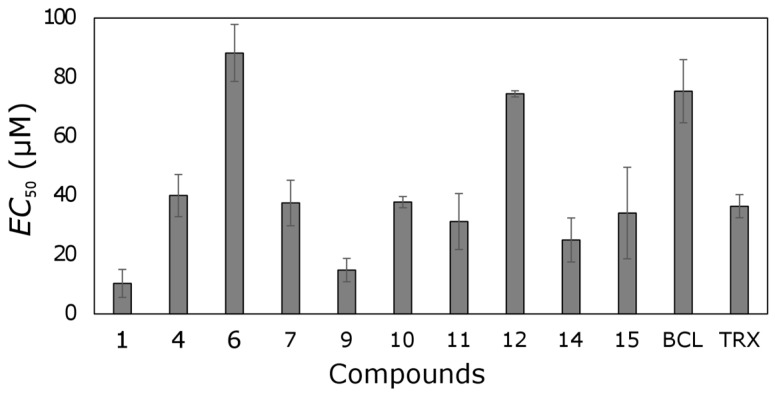
The antioxidant activity of trihydroxyflavones and TRX.

Most tested trihydroxyflavones showed antioxidant activity (Figure 6). Only five out of seventeen compounds did not possess that activity.

Six out of seventeen tested compounds were more active than TRX. Compound **1** possessed the strongest free-radical scavenging effect, and it was 3.5 times more active than TRX. Compound **9** was also one of the most active trihydroxyflavones; its antioxidant activity was 2.5-fold higher than TRX.

Compounds **1** and **9** contain an *ortho*-dihydroxy group in ring B. This structural element together with 3-hydroxy group determines the strong antioxidant activity of compound **1**. The 6-Hydroxy group in ring A of compound **9** led to slightly lower antioxidant effect. Compound **10** also contains *ortho*-dihydroxy groups in ring B, but its activity is two times lower, and the 6-hydroxy group in ring A could contribute to it.

API did not show free radical scavenging activity; its EC_50_ was > 100 µM. Most of the remaining trihydroxyflavones possessed moderate antioxidant activity with EC_50_ values from 20 to 55 µM. 

Cotelle et al. [43] emphasize the importance of the presence of catechol or pyrogalol structural fragments for polyhydroxyflavone antioxidant activity. Trihydroxyflavone, containing the hydroxyl groups in positions 2’, 3’ and 4’ in ring B, exhibited the strongest free radical scavenging ability. Another trihydroxyflavone, containing hydroxyl groups in positions 4’, 5, and 7, possessed a very similar activity as compound **8** examined in this work, having hydroxyl groups in positions 2', 5, and 7. The positions of the hydroxyl groups of both compounds in ring A are the same and neither showed strong antioxidant activity.

Park et al. [44] studied hydroxyflavones containing one, two or three hydroxyl groups. It was established that monohydroxyflavones did not possess strong free radical scavenging properties, whereas several dihydroxy- and trihydroxyflavones showed rather high activity, even higher than vitamin C. However, among the dihydroxyflavones and trihydroxyflavones, there were ones that did not show a strong antioxidant effect. This supports the claim that the location of hydroxyl groups determines the antioxidant activity of hydroxyflavones. In the work of the researcher, the free radical binding capacity of 3′,6,7-trihydroxyflavone was 87.8%. The structure of the trihydroxyflavone considered is the same as that of compound **11** studied in this work. In this work, trihydroxyflavones, containing hydroxyl substitutions in the same positions (4′, 7, 8, and, 3′, 4′, 5), also did not show high antioxidant activity.

Wei et al. [45] determined the antioxidant activity of API and BCL by the DPPH method. It was found that API did not show free radical binding properties, and the BCL EC_50_ was 12.7 ± 0.25 μM. BCL antioxidant capacity does not coincide with the value described in this work (EC_50_ was found to be 80 μM). This can be explained by the influence of pH on the antioxidant activity of hydroxyflavones. It is known that [46] hydroxyflavones have antioxidant properties dependent on pH; deprotonated hydroxyflavones have stronger antioxidant activity.

### 2.3. The Correlation between Trihydroxyflavone Anti-Proliferative and Antioxidant Effect

Based on the calculated Pearson coefficient, no strong correlation was found between the anticancer and antioxidant activity of tested trihydroxyflavones (Table 2).

A moderate correlation was found between the trihydroxyflavone anticancer activity against the A549 cell line and the free radical scavenging properties. Also, there was a moderate correlation between the anti-proliferative effect on glioblastoma cells and antioxidant activity. However, in both cases, those correlations were not proved to be statistically significant (*p* > 0.05).

The anti-proliferative effect on MCF-7 cells and antioxidant activity did not correlate. Those differences between different cell lines could be explained by different mechanisms of action or trihydroxyflavones, and this could be proven by more detailed studies. The established correlation between anti-proliferative effect on A549 and U87 cell lines and antioxidant activity suggests that the anticancer activity in those cells could be related to the antioxidant properties of the tested compounds.

### 2.4. Structure–Activity Relationship

The viability of non-small cell lung cancer cells was most strongly affected by compounds **1**, **3** and **5** containing two hydroxyl groups at positions 3 and 3'. Compound **3** was slightly weaker against the A549 cancer cell line, and the hydroxyl group at position 6 could contribute to this lower activity. Compounds **1**, **7**, and **10** contain two hydroxyl groups in positions 3 and 4 of the B ring. They, together with benzene, form a catechol structural element that could be important for the cell viability reduction activity. In the structure of compound **10**, a hydroxyl group in position 6 of ring A resulted in a weaker anti-proliferative activity. Compounds **2**, **4** and **8**, containing one hydroxyl group in each ring, did not exhibit cell viability reduction. Trihydroxyflavones, containing hydroxyl substitutions at positions 7 and 8, did not reduce the A549 cell viability.

Compound **7**, containing catechol fragment in its structure, was the most active against breast cancer cells. Compound **9** was one of the most potent inhibitors, too. It contains *ortho*-dihydroxy group in ring B, located in positions 1’ and 2’. Compound **3**, the second most active anticancer compound, contains three hydroxyl groups attached to different rings. The other active compounds (EC_50_ ≤ 25 μM) contain two *ortho* hydroxyl groups (compounds **11** and **13**), and compounds **1** and **10** have a catechol structural element. The compounds that were not active against the A549 cell line also did not affect the viability of breast cancer cells, with the exception of compounds **8** and **13** (both having hydroxyl groups at positions 2’ and 7).

The glioblastoma was the least susceptible to the effect of trihydroxyflavones. The importance of the catechol group B ring in the anti-proliferative effect was determined again. This group was presented in compounds **1** and **7**. Compound **3** showed a similar anti-proliferative activity as compound **1**, and was two times more active than compound **7**. It contains all three hydroxyl groups in different positions. Compounds **1** and **3** contain a hydroxyl group in the same position in ring C, and compound **7** contains the third hydroxyl group in ring A, possibly contributing to its lower activity.

The substituents in ring B were the most important for the antioxidant activity of trihydroxyflavones. The strongest antioxidants were compounds **1** and **9**. Compound **1** contains a catechol group, and compound **9** contains two *ortho* hydroxyl groups in ring B. Moderately active antioxidants were compounds **14** and **15**, containing two hydroxyl groups in ring A, and compounds **7** and **10** containing hydroxyl groups in ring B.

**Figure 7:**
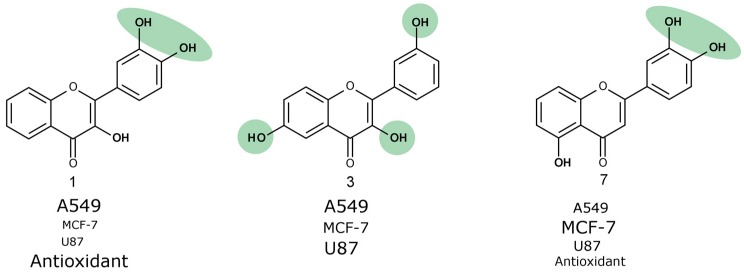
The most active trihydroxyflavones in cancer cell viability and the free-radical scavenging assay in this study. The relative ranking of activity against different cell lines is shown by different font sizes.

The most active trihydroxyflavones against all cancer cell lines and the strongest antioxidants are shown in Figure 7. Compound **7** (3’,4’,5-trihydroxyflavone) was shown to have the highest potency for non-small cell lung cancer (A549) and breast cancer (MCF-7). The viability of glioblastoma cells was most inhibited by compound **3** (3,3′,6-trihydroxyflavone). The most active free radical binding was compound **1** (3,3′,4′-trihydroxyflavone).

Our results showed that the *ortho*-dihydroxy group in ring C is very important for both antioxidant and anticancer activity. However, compound **3** lacking this group and not possessing free radical scavenging properties was also one of the most active anticancer agents. Its anti-proliferation activity could be related to different mechanisms of action.

## 3. Materials and Methods 

### 3.1. Chemicals and Materials

Trihydroxyflavones possessing hydroxyl groups in different positions of 2-phenyl-1,4-benzopyrone (at least 99% pure) were kindly provided by Dr. Vytautas Smirnovas, and originally purchased from Indofine Chemical Company, Inc. (Hillsborough, NJ, USA). The solutions of trihydroxyflavones were made in dimethylsulfoxide (DMSO) directly before experiments. DMSO (≥99%, Ph. Eur. grade) was obtained from Sigma-Aldrich (St. Louis, MO, USA). 6-Hydroxy-2,5,7,8-tetramethylchromane-2-carboxylic acid (Trolox, ≥99% pure) was purchased from Sigma-Aldrich. 2,2-Diphenyl-1-picrylhydrazyl (DPPH, ≥95%) was purchased from Alfa Aesar (Haverhill, MA, USA). 3-(4,5-Dimethylthiazol-2-yl)-2,5-diphenyltetrazolium bromide (MTT, ≥97%) was purchased from Sigma-Aldrich. Ethanol (96.6%) was obtained from Stumbras, LLC (Kaunas, Lithuania).

All cell culture plastic ware was purchased from Thermo Fisher Scientific (Waltham, MA, USA), Corning (Corning, NY, USA) and Techno Plastic Products (Trasadingen, Switzerland). TrypLE^TM^ Express, Dulbecc’o modified Eagle high glucose medium (DMEM Glutamax), fetal bovine serum (FBS), penicillin/streptomycin solution (10,000 U/mL), phosphate buffered saline (PBS) were obtained from Thermo Fisher Scientific.

### 3.2. Cell Culture

Human non-small cell lung carcinoma cell line A549, human breast adenocarcinoma cell line MCF-7 and human glioblastoma cell line U87 (a kind gift from Dr. Manel Esteller, Bellvitge Biomedical Research Institute (IDIBELL)) were grown in DMEM Glutamax medium supplemented with 10% FBS and 1% antibiotics at 37 °C in a humidified atmosphere containing 5% CO_2_. All cell cultures routinely were grown to 70% confluence and trypsinized with 0.125% TrypLE™ Express solution before passage. They were used until passage 20.

### 3.3. Cell Viability Assay

Cell viability was studied using the method of MTT. Cells (100 μL) were seeded in 96-well plates in triplicate (5 × 10^3^ cells/well) and incubated at 37 °C for 24 h. Then, serial dilutions of tested compounds (from 100 µM to 3.125 µM) were made in microplates. Cells treated only with medium containing 0.25% DMSO served as a negative control. Free medium without cells was used as a positive control. After 72 h incubation at 37 °C, 20 μL of MTT 0.5 mg/mL solution in sterile water was added into each well. After 4 hours, the liquid was aspirated from the wells and discarded. Formazan crystals were dissolved in 100 μL of DMSO, and absorbance was measured at a test wavelength of 490 nm and a reference wavelength of 630 nm using a multi-detection microplate reader. The experiments were repeated three times independently, and the results are presented as the means ± SD.

Applying Hill fit to compound dose–cell metabolic activity (absorbance) curves, the effective concentration (EC_50_) values, reducing cell viability by 50%, were calculated.

### 3.4. Antioxidant Activity

The antioxidant activity of trihydroxyflavones was tested by the 2,2-diphenyl-1-picrylhidrazyl (DPPH) radical scavenging method. DPPH 60 µM solution was prepared in 96.6% of alcohol immediately before each experiment and protected from light. 

In a 1-cm diameter quartz cell, 1 mL of ethanolic DPPH solution was mixed with 5 µL of tested trihydroxyflavone solution in DMSO. DPPH ethanolic solution (1 mL) mixed with 5 µL of DMSO served as a negative control. Also, the antioxidant activity of Trolox was established in parallel, and this substance served as a positive control.

Light absorbance was measured at a wavelength of 515 nm using a spectrophotometer: Cary 8454 UV-Vis (Agilent Technologies). Ethanol (96.6%) was used as a blank solution. Experimentally, it has been determined that absorbance constantly decreases and stabilizes after 30 min.; thus, the absorbance of all tested samples was measured 30 min. after the preparation of sample of tested compounds.

Antioxidant activity was evaluated by calculating the effective concentration (EC_50_) at which the tested compound reduced the free radical induced oxidation by 50%. Hill fit to compound dose–antioxidant activity (absorbance) was used.

### 3.5. Statistical Analysis

Data are presented as the mean ± standard error (S.E.) of at least three independent experiments. The correlation between antioxidant and anticancer activity was assessed by calculating Pearson’s coefficient *r* and its statistical reliability. Correlation was considered as very strong when *r* = 0.90–0.99 (positive) or −0.99–(−0.90) (negative); strong when *r* = 0.70–0.89 (positive) or −0.89–(−0.70) (negative); and moderate when *r* = 0.40–0.69 (positive) or −0.69–(−0.40) (negative). Student's t-test was used for comparing two groups. The level of statistical significance was set at *p* < 0.05.

## 4. Conclusions

Trihydroxyflavones are more active against human breast and non-small cell lung cancer cell lines and show lower activity against glioblastoma cells. The majority of trihydroxyflavones show a free radical scavenging effect; some of them are 3.5 times more active than Trolox. The *Ortho*-dihydroxyl structural fragment in ring B is very important for both anticancer and antioxidant activity of trihydroxyflavones. The anticancer effect of trihydroxyflavones against A549 and U87 cells could be related to their antioxidant activity: anti-proliferative effect directly correlates with DPPH radical scavenging activity. 3,3′,6-trihydroxyflavone (contains hydroxyl groups attached to the different rings) does not possess antioxidant activity but is a highly active anticancer compound. It could have different mechanisms of action.

## Figures and Tables

**Table 1:** Anti-proliferative effect of trihydroxyflavones. White color—EC_50_ >50 µM, light grey—EC_50_ <50 µM, dark grey—EC_50_ <25 µM.

Compound	EC_50_ (µM)		
	A549	MCF-7	U87
**1**	19.6 ± 4.5	21.6 ± 3.6	15.5 ± 5.4
**2**	>100.0	>100.0	>100.0
**3**	36.3 ± 4.6	12.6 ± 2.6	14.2 ± 4.8
**4**	>100.0	>100.0	>100.0
**5**	19.8 ± 8.3	36.0 ± 0.3	72.6 ± 4.6
**6**	75.3 ± 5.7	69.3 ± 3.5	80.4 ± 6.8
**7**	6.8 ± 0.6	11.2 ± 1.1	30.8 ± 2.8
**8**	>100.0	30.0 ± 0.6	59.8 ± 8.7
**9**	22.1 ± 2.0	16.2 ± 4.2	>100.0
**10**	37.6 ± 2.7	19.0 ± 5.7	59.5 ± 3.8
**11**	69.2 ± 0.6	23.1 ± 5.5	88.4 ± 14.4
**12**	>100.0	>100.0	>100.0
**13**	>100.0	15.9 ± 4.3	>100.0
**14**	>100.0	>100.0	>100.0
**15**	>100.0	71.5 ± 13.6	73.8 ± 13.6
API	77.5 ± 9.2	71.5 ± 15.6	>100.0
BCL	68.2 ± 3.6	26.1 ± 7.1	>100.0

**Table 2:** The correlation between trihydroxyflavone anti-proliferative and antioxidant activity

Cell Line	*r*	*p*
A549	0.45	0.07
MCF-7	0.18	0.49
U87	0.43	0.09
